# Implementation and Optimization of Zero-Knowledge Proof Circuit Based on Hash Function SM3

**DOI:** 10.3390/s22165951

**Published:** 2022-08-09

**Authors:** Yang Yang, Shangbin Han, Ping Xie, Yan Zhu, Zhenyang Ding, Shengjie Hou, Shicheng Xu, Haibin Zheng

**Affiliations:** 1 School of Cyber Science and Technology, Beihang University, Beijing 100191, China; 2 National Innovation Institute of Defense Technology, Academy of Military Sciences, Beijing 100071, China; 3 Neusoft Corporation, Shenyang 110179, China; 4 Liaoning Blockchain Engineering Technology Research Center, Shenyang 110179, China; 5 Hangzhou Innovation Institute, Beihang University, Hangzhou 310051, China

**Keywords:** SM3, zero-knowledge proof, arithmetic circuit, privacy preserving

## Abstract

With the increasing demand for privacy protection in the blockchain, the universal zero-knowledge proof protocol has been developed and widely used. Because hash function is an important cryptographic primitive in a blockchain, the zero-knowledge proof of hash preimage has a wide range of application scenarios. However, it is hard to implement it due to the transformation of efficiency and execution complexity. Currently, there are only zero-knowledge proof circuits of some widely used hash functions that have been implemented, such as SHA256. SM3 is a Chinese hash function standard published by the Chinese Commercial Cryptography Administration Office for the use of electronic authentication service systems, and hence might be used in several cryptographic applications in China. As the national cryptographic hash function standard, the zero-knowledge proof circuit of SM3 (Chinese Commercial Cryptography) has not been implemented. Therefore, this paper analyzed the SM3 algorithm process, designed a new layered circuit structure, and implemented the SM3 hash preimage zero-knowledge proof circuit with a circuit size reduced by half compared to the automatic generator. Moreover, we proposed several extended practical protocols based on the SM3 zero-knowledge proof circuit, which is widely used in blockchain.

## 1. Introduction

With the gradual maturity of emerging technologies such as blockchain and cloud computing, people’s new demands for sensitive data protection and the development of privacy protection technologies promote each other. Admittedly, zero-knowledge proof technology is one of the most concerned academia and industry areas of research in recent years. As an important cryptographic primitive, hash function has a wide range of application scenarios combined with zero-knowledge proof.

Zero-knowledge proof is an efficient protocol based on probabilistic verification, which enables the prover to prove to the verifier that the prover knows a secret value without revealing any information about the value. As early as 1987, it was proved that any NP (non-deterministic polynomial) problem has a zero-knowledge proof [1]. The general solution for converting NP problems into zero-knowledge proofs is to split the problem into an intermediate form expressed by arithmetic gates or logic gates as the basic operation, and then extract the intermediate form into a circuit description matrix; finally, the proof is generated based on the back-end program of a specific zero-knowledge proof protocol.

However, using a unified automatic zero-knowledge proof circuit generator for different algorithms cannot meet the current high efficiency requirements. The zero-knowledge proof of hash function is increasing in practical application scenarios of blockchain, and more efficient algorithms are required for circuit conversion. At present, the zero-knowledge proof circuit based on SM3 has not yet been implemented. It needs an efficient circuit implementation method as a crucial zero-knowledge proof algorithm.

The main research content of this paper is the design and implementation of a zero-knowledge proof circuit based on the typical hash function SM3. At the current stage of zero-knowledge proof development, the main implementation difficulty lies in converting the computational process of the problem to be proved into an arithmetic circuit, which is a tedious and low-fault-tolerant task. Starting from the corresponding relationship between the calculation steps of SM3 and the circuit modules, we designed a circuit-layered structure. Each layer contains multiple zero-knowledge proof sub-circuits, and is composed of lower-level sub-circuits. With the rational design of the circuit structure and the normalization of the circuit generation paradigm, we implement a secure and efficient SM3 zero-knowledge proof circuit and extend a variety of zero-knowledge proof protocols on this basis. The main contributions of this paper are as follows.

1. A layered structure of SM3 circuit is proposed, which realizes circuit decoupling and multiplexing, and the lower-layer circuit is transparent to the upper-layer circuit. The circuits designed through this architecture have low coupling, and each packaged sub-circuit has a specific logical meaning, which is convenient for later expansion of larger circuits.
2. We implemented the SM3 zero-knowledge proof circuit and performed functional and performance tests on the physical machine. Full conversion details and paradigms are given for each subcomputing step of SM3 to the R1CS circuit-constrained form. The realized SM3 hash preimage zero-knowledge proof circuit has high efficiency, and its complexity is reduced by half compared with the general zero-knowledge proof circuit generator. The realization effect is comparable to the current optimal implementation of SHA256.
3. Based on the realized SM3 zero-knowledge proof circuit, various zero-knowledge proof protocols are extended and implemented. We designed and implemented zero-knowledge proof circuits for an elliptic curve discrete logarithm and SM3 preimage equality, hash chain proof, and finally tested the implementation results.

This paper is organized as follows. In Section 2, preliminary knowledge related to the SM3 zero-knowledge proof circuit in this article is introduced. We give the architecture design of the zero-knowledge proof circuit based on SM3 and analyze how the SM3 zero-knowledge proof circuit is generated in Section 3. Then, in Section 4, we study a detailed circuit conversion generation scheme and the dependencies between the circuits. Additionally, a specific SM3-based zero-knowledge proof circuit conversion generation paradigm is introduced. In Section 5, we implement the results of functional and performance tests and analyze the test results. In Section 6, we propose several concrete implementations extend practical protocols based on SM3 zero-knowledge proof circuit, which are widely used in blockchain. Finally, we conclude this paper in Section 7.

## 2. Related Work

In 2016, Groth et al. [2] proposed a concise, non-interactive, zero-knowledge proof scheme based on bilinear pairings, which is called Groth16. The most significant advantage of this scheme is that it uses asymmetric pairing, so that the proof only contains three group elements; as a result, the new system cannot surpass it in proof size in recent years. However, Groth16 requires a trusted setup and re-runs the initialization phase with different parameters in the face of other circuits, which causes specific difficulties for its practical use. Based on the inner product proof technique proposed by Bootle et al. [3], Bünz et al. [4] proposed the Bulletproofs zero-knowledge proof system in 2017. Bulletproofs supports logarithmic-sized aggregate proofs and does not require trusted initiation. Sasson et al. [5] proposed zk-STARKs in 2018, a quantum-attack-resistant zero-knowledge proof system without trusted guidance. Maller et al. [6] continued to optimize the globally updatable CRS in 2019, reducing the size of the CRS from the square level of the circuit size to a linear level.

Admittedly, the most cumbersome part of the general zero-knowledge proof scheme is expressing the problem in the form of an arithmetic circuit or a Boolean circuit. In 2010, Meiklejohn et al. [7] implemented a zero-knowledge proof description language system, which has an interpreter that can convert the input program into a special cryptographic language. After that, such zero-knowledge proof circuit representation tools began to develop. For example, Sasson et al. [8] proposed a virtual machine called TinyRAM in 2011, which can receive NP problems described in C language and execute the proof and verification process in the virtual machine. In 2013, Parno et al. [9] provided a compiler that can convert C language program into quadratic arithmetic and quadratic span programs. The team continued to improve TinyRAM, and proposed new models vnTinyRAM and scalable TinyRAM. The scalable TinyRAM refers to the idea of recursive proof generation by Valiant et al. [10] and Bitansky et al. [11], and realizes recursive proof generation through elliptic curve technology.

The toolchain Pinocchio provides zero-knowledge proof system a near-practical standardized solution, and is still in use today. In addition, there is also another typical zero-knowledge proof circuit generator Pentry proposed by Braun et al. [12]. Pentry enables computational processes to be stored on untrusted memory, mainly by the prover generating a digest for each computational process. Given the efficiency of automatic circuit generation, Kosba et al. [13] developed a new compiler xJsnark which optimizes short and extended integer types and supports programming through JAVA. Dimitris et al. [14] present Zilch, a framework that accelerates and simplifies the deployment of VC and ZKPK for any application transparently, i.e., without the need of trusted setup. Although the current zero-knowledge proof arithmetic circuit generation tools can automatically perform the conversion from calculation to circuit, the conversion efficiency is low, which is also its future optimization direction.

## 3. The Preliminaries

### 3.1. Related Cryptography Technology

#### 3.1.1. Hash Function

The hash function *H* takes a variable-length data block *M* as input, and generates a fixed-length hash value h=H(M). The hash function [15] can guarantee the integrity of the message, and any change in the data block *M* will greatly change its hash value. Informally, a cryptographic hash function has to fulfill the following security requirements:

1. Collision resistance: it is practically infeasible to find two messages *M* and $M^{*}$, with $M\neq M^{*}$, such that $H\left(M\right)=H\left(M^{*}\right)$;
2. Second preimage resistance: for a given message *M*, it is practically infeasible to find a second message $M\neq M^{*}$ such that $H\left(M\right)=H\left(M^{*}\right)$;
3. Preimage resistance: for a given hash value *h*, it is practically infeasible to find a message *M* such that H(M)=h.

The hash function can be divided into two parts, namely the iterative structure and the compression function. The iterative structure can split the input message into multiple message blocks and compress them separately, so that the hash function can process the message if it is arbitrarily long. The compression function compresses the current message block into a seemingly random value of fixed length through diffusion and obfuscation.

**Figure 1:**
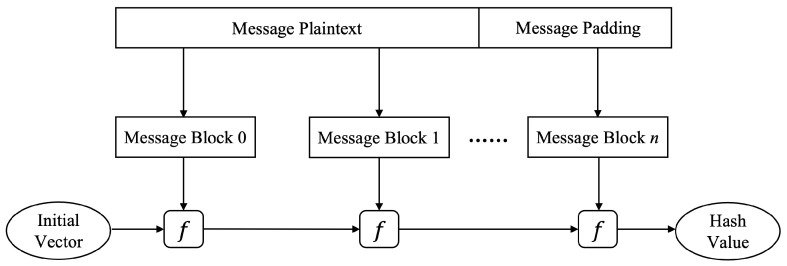
The basic construction of Merkle–Damgård structure in hash function.

At present, the iterative structure of hash function can be mainly divided into two types: Merkle–Damgård structure and sponge structure. The Merkle–Damgård structure [16] has a suitable property; if its compression function is collision resistant, then the hash function with this structure is also collision resistant. Early hash functions such as MD5, SHA-1, and SHA256 adopted this design. The Merkle–Damgård structure is shown in Figure 1.

**Figure 2:**
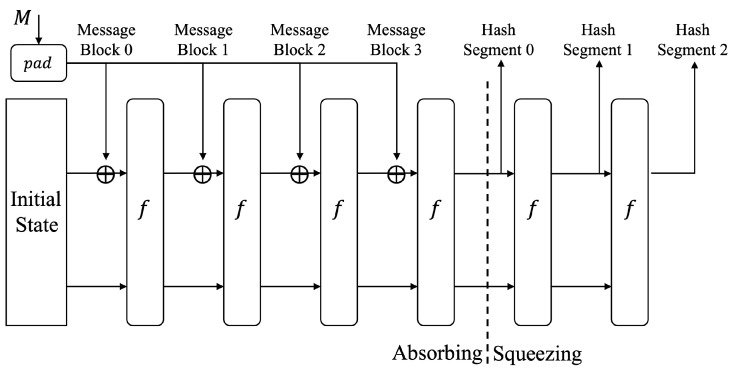
The basic construction of Sponge structure in hash function.

Sponge structure [17] has been a flexible and efficient hash function construction method in recent years. Hash functions such as SHA-3 and Poseidon are designed based on this structure. It divides the processing of data into two parts: absorbing and squeezing. The absorbing part inputs the message in chunks; then outputs the hash result in the squeezing step. The sponge structure is shown in Figure 2.

#### 3.1.2. Discrete Logarithmic Cryptosystem

For public-key cryptography, it can be divided into three categories: a public-key encryption RSA cryptosystem, discrete logarithm cryptosystem, and an elliptic curve cryptosystem. Among them, RSA encryption is based on a large integer factorization problem, and the latter two are based on a discrete logarithm problem. A discrete logarithmic cryptosystem will depend on the following condition; that is, there exists some abstract prime *p*-order multiplicative group *G*, and the primitive root of *p* is *g*, satisfying:

1. It has an efficient and achievable fast modular exponentiation algorithm; that is, it can quickly calculate $g^{n}\;\mathrm{mod}\;p$;
2. It is difficult to calculate $\log_{g}g^{n}\;\mathrm{mod}\;p$, and no polynomial time algorithm has been found to solve it.

#### 3.1.3. Hash Chain

Hash chain is a crucial application based on hash function implementation in cryptography, which can be used to generate one-time keys or to force serial calculation scenarios. For the hash function *h* and the input message *x*, a hash chain with a length of *n* means that the calculation result of h(x) is used as the following input message, and it is recursively calculated *n* times, usually denoted as $h^{n}\left(x\right)$. Hash chains have two characteristics:

1. If the hash function *h* is one-way, then the hash chain is also one-way;
2. The calculation of the hash chain is serialized, and it is impossible to calculate multiple hash functions in parallel, nor to start the calculation from the non-head position of the hash chain.

Based on unidirectionality, Park et al. [18] proposed using hash chains to generate one-time keys in weakly secure environments. When the server needs to provide authentication services, it does not directly use the plaintext of the password, but uses a hash chain for verification to prevent the password from being stolen and used directly during the transmission process.

#### 3.1.4. Merkle Tree

Merkle tree was proposed by Merkle et al. [19] in 1987. It is also known as hash tree, and can efficiently verify the integrity of a particular datum in a set of data. Merkle tree is usually implemented based on binary tree, and can also be implemented based on multi-fork tree. The value of each non-leaf node is the hash value of the value of the direct child node of the node, and the value of its root node is the root hash. Merkle tree has been widely used in many peer-to-peer applications, including the interplanetary file system IPFS [20], Zeronet [21], version control system Git [22] and so on.

For a Merkle binary tree of depth *D*, it can hold $2^{\left(D-1\right)}$ leaf nodes. When verifying the data integrity of a data block, only 2D−1 hash values need to be provided, and the hash value is calculated *D* times. Therefore, the verification complexity of a Merkle tree is only O(logN) compared to the O(N) time complexity of verifying the hash list, where *N* is the number of data blocks.

### 3.2. Zero-Knowledge Proof Circuit

The zero-knowledge proof circuit is an important concept, and a key step of the general zero-knowledge proof protocol. The general zero-knowledge proof protocol [23] is based on circuit satisfiability, which can transform any NP problem into a zero-knowledge proof. Zk-SNARKs [24] is the most widely used class of general-purpose zero-knowledge proof protocols, and its letters mean:

1. zk: Zero-knowledge—the prover does not reveal any information about the secret;
2. S: Succinct—the length of the proof is less than the length of the evidence, and the evidence information cannot be extracted through the proof;
3. N: Non-interactive—the proof process is non-interactive, there is no need for multiple interactions between the prover and the verifier, and only one proof can be publicly verified;
4. ARK: Argument of Knowledge—the proof is computationally unforgettable.

For any calculation, there are input values and output values. When the prover knows an input value that makes the calculation result in a specific output value, he must know any intermediate value of the calculation process. To facilitate computer processing, the calculation process can be converted into a unified representation form of a Boolean circuit or an arithmetic circuit. The two input values and one output value corresponding to each circuit gate need to satisfy corresponding constraints. All gate constraints of the converted circuit can be described in the form of R1CS matrix. Through this matrix, the corresponding proof polynomial can be generated, and zk-SNARKs can be proved and verified according to the properties of probabilistic polynomial verification.

The part that needs to be implemented manually in zero-knowledge proofs is converting the computation into the circuit constraints expressed in matrix form. This paper improves the circuit conversion process of the SM3 algorithm, reduces the number of intermediate variables and circuit gates manually, and improves the conversion efficiency of the algorithm to the zero-knowledge proof circuit. Taking the function $y=x^{2}+x$ as an example, according to the priority of the operation symbol, the calculation process of the function is divided into multiple additions or multiplication operations in sequence, and the result of each binary operation is stored in a new intermediate variable:

(1) $$\begin{array}{c} v_{0}=x\cdot x\\ y=v_{0}+x \end{array}$$

$v_{0}$ represents the intermediate variable of the calculation process, and each binary operation can be instantiated as an addition or multiplication circuit gate. The variables correspond to two input pins and one output pin of the circuit gate.

The microscopic significance of zero-knowledge proof is to prove that the equality sign of each intermediate operation is established, so these variables need to be constrained according to the corresponding circuit gates. This step is called R1CS constraint, which uniformly constrains the input and output of all the addition and multiplication operation gates of the calculation circuit, and uses three matrices to represent all constraint parameters, to make good use of the matrix calculation capability of the computer.

### 3.3. Hash Preimage Zero-Knowledge Proof

The hash preimage zero-knowledge proof is defined as ZK{x|y=H(x)}. For the publicly available hash function *H* and hash value *y*, the prover proves that it knows the input message *x*, such that y=H(x). The equation holds without revealing other information about the message preimage *x*. A cryptographically secure hash function has good one-way and collision resistance, so its preimage cannot be deduced from the hash value of a cryptographically secure hash function. Since a hash value can be generated from an input message of arbitrary length, in some application scenarios, proving that the preimage of a specific hash value is known is proof that the corresponding message is possessed. In many application scenarios of blockchain and cloud computing, it is necessary to generate proofs without revealing the message preimage, so the zero-knowledge proof of the hash preimage is of great significance. At present, some computing frameworks in the blockchain field, such as Zokrates [25] and ZkBoo [26], provide implementation of preimage proof for specific hash functions.

Since the hash function does not have an excellent algebraic structure, it can only be calculated in a general zero-knowledge proof method, and the calculation process is converted into a circuit constraint form. The size and depth of the circuit will determine the size and speed of the proof. As the most widely used hash function in cryptography, SHA256 has multiple versions of hash preimage zero-knowledge proof implementations. Multiple cryptocurrencies use SHA256 to verify transactions and for proof of work [27] or proof of stake [28]. However, its circuit size is large, and the proof efficiency is low. To this end, many SNARK-friendly hash functions have been invented and implemented, such as MiMC [29] and Poseidon [30]. At present, there is no implementation of hash preimage zero-knowledge proof based on SM3. This paper solves the vacancy of the SM3 algorithm, manually optimizes the circuit conversion of the algorithm, and dramatically improves the efficiency of hash preimage zero-knowledge proof based on SM3.

### 3.4. Typical Hash Function

#### 3.4.1. SM3

The National Secret SM3 hash function is one of the commercial cryptographic systems released by the State Cryptography Administration [31] in December 2010. SM3, whose security is comparable to SHA256 [32], is mainly used for digital signature and verification, message authentication code generation and verification, random number generation, etc. Compared with SHA256 [33], the complexity of SM3 is relatively higher, and each round of compression function introduces two message words.

**Figure 3:**
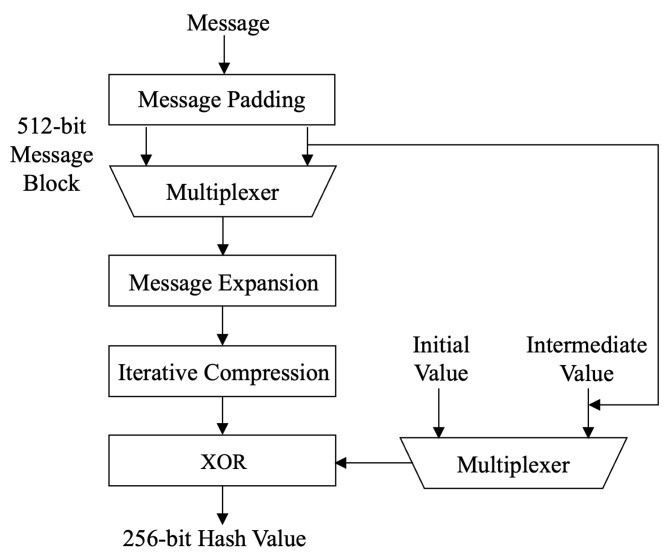
The calculation process of SM3.

For a message *m* whose length is less than 512 bits, the SM3 hash algorithm is padded and iteratively compressed to generate a hash value with a length of 256 bits, as shown in Figure 3. The specific process includes the following four steps.

1. Message padding: In this step, we pad the message length to an integer multiple of 512 so that the input message can be processed by the compression function in units of 512 bits;
2. Initialization buffer: The buffer is used to save the intermediate and final results of the SM3 function, which is represented by eight 32-bit registers: A, B, C, D, E, F, G, and H. These registers are initialized to the 32-bit message words: A = 7380166f, B = 4914b2b9, C = 172442d7, D = da8a0600, E = a96f30bc, F = 163138aa, G = e38dee4d, H = b0fb0e4e;
3. Message iterative compression: This step is its core part, compressing the input register variable and 512-bit message block into a 256-bit random output through Boolean and algebraic operations. For the compression function CF, there is V(i+1)=CF(V(i),B(i)), where *V* represents the register value and *B* represents the message block. CF contains 64 steps of iterative operations. Each step takes registers A, B, C, D, E, F, G, and H as inputs and updates itself through calculation. Each iteration uses the 32-bit long $W_{j}$ and $W_{j}^{{}^{\prime }}$, which are iteratively generated by the message packet B(i) according to the message expansion rules. The main operations involved in the compression function include:
  (2) $$SS1\leftarrow (\left(A\ll 12\right)+E+\left(T_{j}\ll J\right))\ll 7$$
  (3) SS2←SS1⨁(A≪12)
  (4) $$TT1\leftarrow FF_{j}\left(A,B,C\right)+D+SS2+{W_{j}}^{\prime }$$
  (5) $$TT2\leftarrow GG_{j}\left(E,F,G\right)+H+SS1+W_{j}$$
  Among them, $FF_{j}\left(\cdot \right)$ and $GG_{j}\left(\cdot \right)$ are two Boolean function operations, and $T_{j}$ is a constant. At the same time, to improve the diffusion rate, the P0/P1 replacement function is introduced, which achieves the advantages of fast diffusion speed and good diffusion effect;
4. Hash value output: After all 512-bit packets are processed, the output of the last 512-bit packet is the message digest value.
  The message expansion and compression functions are the most computationally intensive part of the SM3 execution process, and are also the most critical part of designing the circuit structure, which determines the generation efficiency of the circuit.

#### 3.4.2. Poseidon

With the development of zero-knowledge proof technology, more and more zero-knowledge-proof-related applications based on hash functions have been proposed. In order to reduce the size of the corresponding circuit of the hash function and improve the efficiency of zero-knowledge proof, a class of hash functions called SNARK-friendly hash was created. Poseidon is the most recently designed and most efficient SNARK-friendly hash function. It uses a sponge structure to process arbitrarily long message blocks and can output arbitrarily long digests as needed. Its most significant advantage is that it adopts an efficient S-box design, such as using $x^{3}$ or $x^{5}$, which reduces the circuit size by two orders of magnitude with the same security parameters as SHA256.

Based on the high efficiency and easy implementation of Poseidon, many blockchain projects have adopted this hash algorithm, such as Filecoin’s [34] Merkle tree proof, Loopring’s private transaction on the second layer of Ethereum, etc.

## 4. Circuit Hierarchical Architecture Design Based on SM3

This section mainly introduces the design ideas of the entire SM3 circuit architecture. First, we determine the zero-knowledge proof framework after implementing the circuit program. Then, the specific program function modules in the circuit implementation are introduced from the horizontal level. Finally, we divide the circuit scale and calling relationship into layers, showing the circuit architecture design at the vertical level.

### 4.1. Frame Selection

The generation process of zero-knowledge proof is similar to the principle of the compiler. It can be divided into two parts: front-end and back-end. The front-end is responsible for converting the problem that needs to be proved into a form that can be accepted by the zero-knowledge proof protocol, such as arithmetic circuits, etc., while the back-end of zero-knowledge proof generation is responsible for generating zero-knowledge proofs for this specific intermediate form, and indicators such as generation efficiency and security will depend on which zero-knowledge proof protocol the back-end adopts.

Zero-knowledge proof circuit generation is the process of zero-knowledge proof front-end generating an intermediate form of zero-knowledge proof. The general solution to this process is to use circuit generation tools such as Pinocchio, which is similar to the front-end of a compiler. It automatically converts each intermediate variable during statement execution into pin variables of an arithmetic circuit by analyzing the syntax of high-level language statements, and finally generates a complete circuit. However, this automated tool creates a lot of circuit redundancy and reduces the proof efficiency. By manually generating the SM3 circuit, we rationally design the circuit conversion structure, improve the reusability of the sub-circuit structure, and ultimately improve the conversion efficiency and ensure the correctness of the circuit.

A more suitable back-end framework can be selected by analyzing the efficiency of several zero-knowledge proof protocols based on arithmetic circuits. Table 1 shows that in terms of proof generation time, in addition to zk-STARKs requiring complexity of O(npolylogn), the time complexity of several other zero-knowledge proof schemes is better, which is O(nlogn). Moreover, Groth16, Sonic, and Plonk can achieve a constant level in proof size, but the proof size of Bulletproofs and zk-STARKs will increase with the logarithmic or logarithmic square speed with the circuit size.

Compared with range proof, membership proof, etc., SM3 preimage proof has multiple rounds of round-robin operation and a larger number of bits of operation data, resulting in enormous circuit size. Therefore, zero-knowledge proof schemes such as Groth16, Plonk, etc., whose proof efficiency has relatively low asymptotic complexity relative to circuit size, will be considered first.

At the same time, it is also necessary to consider the development ecosystem of specific zero-knowledge proof circuits. The libsnark library based on Groth16 is currently the most widely used zero-knowledge proof library, which has a rich built-in circuit library. Developing in C++ makes it a good ecosystem, and it is easy to combine with others’ open-source circuit codes. The purpose of this implementation of SM3-based hash preimage zero-knowledge proof is to allow more application scenarios for state secrets in zero-knowledge proof, so the follow-up application research and development based on this circuit is essential. Therefore, the ecology and ease of use of libsnark give it a great advantage.

### 4.2. Functional Module Design

From the horizontal level, the architecture of this paper can be divided into the following functional modules.

The variable assignment module assigns each variable of the SM3 algorithm to the zero-knowledge proof circuit carrier object of libsnark, where the variables can be divided into public and private variables. The public variable refers to the public input in the proof, such as the image in the SM3 known preimage proof, while the private variable refers to the secret input in the proof and the intermediate value generated in the calculation process. In addition, the operation process is divided into modulo operation and bit operation. The bit operation needs to split the variable into multiple bits for allocation. When a variable is used numerous times in the calculation, it should not be assigned multiple times, which will cause redundancy of variables and repeated constraints between the same variables.

The variable constraint module generates R1CS constraints from the circuit variables assigned by the previous module, i.e., the values represented by each circuit gate’s input and output pins. Multiple related R1CS constraints can be combined linearly, and the variables generate constraints of the form (A,X)∗(B,X)=(C,X). This is also the most critical step; namely, a complete arithmetic circuit structure is generated. *A*, *B* and *C* are the matrices that fully represent the constraint coefficients of the circuit, and *X* represents the vector formed by the variables in the circuit. Only the correct *X* can make this equation hold.

The evidence generation module computes and assigns the *X* vectors in the constraints generated by the previous module. The assignment of *X* as a variable that makes the equation true is called the witness in zero-knowledge proofs. The module first assigns each input variable, and then disassembles each calculation step of the entire calculation circuit. It assigns values to intermediate variables step by step, and finally generates complete evidence that can satisfy the circuit constraints.

### 4.3. Circuit Hierarchy Design

The architecture design of the vertical layer of the SM3 zero-knowledge proof circuit is divided into layers based on circuit scale and calling relationship. After the low-level circuit modules are encapsulated, low-level modules can be directly contacted by high-level modules, and the lower layer is transparent to the upper layer. Its four-layer structure is divided from bottom to top into:

1. The auxiliary operation layer: This layer provides the minimum unit circuit required for this design and can realize basic arithmetic circuits such as bit operation and format conversion. All upper-layer circuits are constructed based on these auxiliary circuits;
2. The core operation layer: This layer provides the most core Boolean function and the arithmetic circuit of the permutation function in the SM3 compression function. The compression function of SM3 involves multiple rounds of iterative operations, and each round of iterative operations involves a large number of Boolean functions and permutation functions in this layer. Therefore, the efficiency of circuit conversion in this layer will significantly affect the final circuit size;
3. The iterative compression layer: This layer implements the complete circuit of the SM3 compression function to compress a single message block, which can be divided into the message expansion circuit and the argument circuit. This layer implements the basic computing unit of SM3;
4. Merkle–Damgård layer: This layer connects the circuits of the iterative compression layer in series, thereby realizing the calculation of the SM3 hash value for any length of message input and generating circuit constraints.

**Figure 4:**
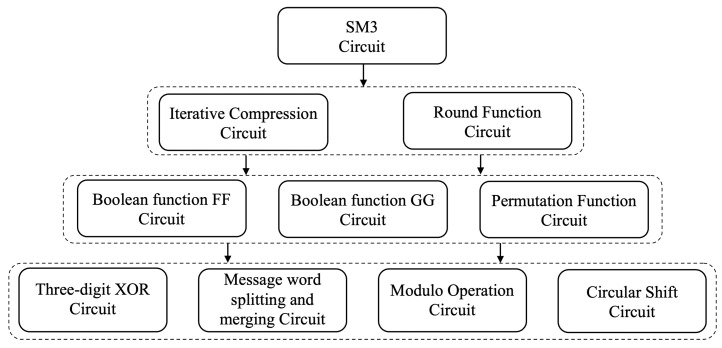
The four-layer structure of SM3 from bottom to top.

The vertical layer design of the circuit is shown in Figure 4, and each layer of circuit is composed of several sub-circuit modules.

#### 4.3.1. Auxiliary Operation Layer

- Three-digit XOR Module

This XOR3 module generates a two-bit or three-bit XOR circuit.

- Message word splitting and merging module

When there are two forms of a single message word and a bit operation variable in the variable operation, this packing module is used to constrain a single message word variable and 32-bit variables. It is applied in Scenarios in which bit operations and modulo $2^{32}$ addition operations are alternately performed, such as the computation of TT1 and TT2 variables in a compression function.

- Mod Reduce Module

The function of the circuit constraint generated by the modulo operation is to ensure that the non-modulo variable and the modulo variable are consistent in the useful bits. The function of the circuit constraint generated by the modulo operation is to ensure that the non-modulo variable and the modulo variable are consistent in the useful bits. Therefore, it is necessary to use the message word splitting circuit above to establish constraints on the non-modulus variables and multiple bit variables, and then use the message word merging circuit to establish constraints on the lower 32-bit bit variables and the modulo variables, and thus establish constraints before and after the mold.

- Circular shift Module

This circular shift module cyclically shifts the array of bit variables to the left without changing the content represented by a single bit variable, and consequently, it does not affect other bit operations.

#### 4.3.2. Core Operation Layer

- FF Module

The following Boolean function FF is used in the compression function of SM3.

(6) $$FF_{j}\left(A,B,C\right)=\left\{\begin{array}{ccc} \; & X⨁Y⨁Z, & 0\leq j\leq 15,\\ \; & (X\wedge Y)\vee (X\wedge Z)\vee (Y\wedge Z), & 16\leq j\leq 63. \end{array}\right.$$

- GG Module

The following Boolean function GG is used in the compression function of SM3.

(7) $$GG_{j}\left(A,B,C\right)=\left\{\begin{array}{ccc} \; & X⨁Y⨁Z, & 0\leq j\leq 15,\\ \; & (X\wedge Y)\vee (\neg X\wedge Z), & 16\leq j\leq 63. \end{array}\right.$$

- Permutation function module

After two cyclic shifts, the permutation function performs a 32-bit XOR operation on a message word and its value. SM3 uses the following two permutation functions.

(8) $$P_{0}\left(X\right)=X⨁\left(X\ll 9\right)⨁\left(X\ll 17\right)$$

(9) $$P_{1}\left(X\right)=X⨁\left(X\ll 15\right)⨁\left(X\ll 23\right)$$

#### 4.3.3. Iterative Compression Layer

- Message Schedule Module

Message expansion refers to expanding the 16 words divided by the message group into 132 words through two rounds of circulation.

$W_{1},\cdots ,W_{67}$ is generated as follows.

(10) $$W_{j}=P_{1}\left(W_{j-16}⨁W_{j-9}⨁\left(W_{j-3}\ll 15\right)\right)⨁\left(W_{j-13}\ll 7\right)⨁W_{j-6}$$

${W_{1}}^{\prime },\cdots ,{W_{67}}^{\prime }$ is generated as follows.

(11) $${W_{j}}^{\prime }=W_{j}⨁W_{j+4}$$

- Round Function Module

The input of SM3 compression function is the output of the previous round of compression function and the message word after the block expansion of the current message. This module performs 64 rounds of iterative operations on these two data, uses A, B, C, D, E, F, G, and H registers to store intermediate variables, and finally obtains the 256-bit output of the current compression function. The complete calculation process is as follows, shown in Algorithm 1.

#### 4.3.4. Merkle–Damgård Layer

This layer groups the message input instantiates multiple iterative compression circuit modules according to the grouping situation, and connects them in series. As the private input of the proof, the hash preimage is passed into the message input part of each iterative compression circuit. Correspondingly, as the public input, the hash value is assigned to the output circuit pin of the last iterative compression circuit.
**Algorithm 1** SM3 round function algorithm**Input**  A, B, C, D, E, F, G, H register initial value **Output**  A, B, C, D, E, F, G, H register update value
 1:** function** ROUNDFUCTION (A,B,C,D,E,F,G,H)
 2:    i←0 3:    **while** i<64 **do** 4:        $SS1\leftarrow (\left(A\ll 12\right)+E+\left(T_{j}\ll J\right))\ll 7$ 5:        SS2←SS1⨁(A≪12) 6:        $TT1\leftarrow FF_{j}\left(A,B,C\right)+D+SS2+{W_{j}}^{\prime }$ 7:        $TT2\leftarrow GG_{j}\left(E,F,G\right)+H+SS1+W_{j}$ 8:        D←C 9:        C←B≪9 10:        B←A 11:        A←TT1 12:        H←G 13:        G←F≪19 14:        F←E 15:        $E\leftarrow P_{0}\left(TT2\right)$ 16:        i←i+1 17:    **end while** 18:     **return** A,B,C,D,E,F,G,H 19: **end function**


### 4.4. Security Analysis

For the zero-knowledge proof protocol, its security consists of three parts: completeness, soundness, and zero knowledge. Here, we combine its completeness and soundness as correctness and analyze it separately from zero-knowledge.

Verifying zero-knowledge proof requires a probabilistic algorithm, which essentially challenges all circuit gate constraints of the zero-knowledge proof circuit. Only if all R1CS constraints for the computational transformation are correct can it be guaranteed that all intermediate variables will satisfy the constraints when the input evidence is valid.

The circuit hierarchy design in this paper performs the conversion of all calculation steps according to the SM3 standard algorithm. These conversions can be divided into Boolean operation conversion, algebraic operation conversion, and mixed operation conversion. Therefore, the design ensures the correctness of constraint generation from two dimensions of the calculation process and the calculation form.

Zero-knowledge should guarantee that the circuit-generated proofs contain information independent of the circuit’s secret inputs. This property depends on the zero-knowledge proof back-end protocol. Since the Groth16 based on the libsnark framework selected in this design is statistical zero-knowledge, this property can be satisfied.

As described, this circuit is secure in theoretical design, and its correctness can be further verified in the functional test in Section 4.

## 5. Implementation and Optimization of SM3 Preimage Zero-Knowledge Proof Circuit

### 5.1. Circuit Conversion

This section converts the specific operations of each step of SM3 into addition gates and multiplication gates and generates the R1CS constraint relationship.

#### 5.1.1. Auxiliary Operation Circuit

- Message word splitting and merging circuit

The compression function of SM3 includes not only algebraic operations, such as modulo $2^{32}$ addition, but also various Boolean operations, such as FF and GG Boolean functions. Algebraic operations use a 32-bit message word as the basic operation unit, while Boolean operations operate on a per-bit basis. Therefore, for mixed operations involving algebraic and Boolean operations, the 32-bit message word needs to be continuously converted between a single digit and a 32-bit existence. For example, for message word *X*, its binary representation is $x_{n}x_{n-1}\cdots x_{0}$. Its algebraic form and the equation relationship between each bit based on addition and multiplication are:

(12) $$X=2^{n}x_{n}+2^{n-1}x_{n-1}+\cdots +x_{0}$$

Furthermore, the three variable terms of its R1CS constraint are:

(13) $$A=2^{n}x_{n}+2^{n-1}x_{n-1}+\cdots +x_{0}$$

(14) B=1

(15) C=X

In addition to the constraint between a single bit and a complete message word, there is an implicit constraint. Since there is no data type for variables in the circuit, additional constraints need to be placed on the variables representing a single bit to ensure its ’bit character’; namely, any bit *x* after the expansion of the message word should satisfy:

(16) x(x−1)=0

The RICS constraint is:

(17) A=x

(18) B=x

(19) C=x

- Three-digit XOR circuit

The circuit is implemented based on the combination of three single-bit numbers. For the single-bit Boolean operation result=x⨁y⨁z, it can be divided into two steps, where result is the calculation result, and aux is the calculated intermediate value:

(20) aux=x⨁y

(21) result=aux⨁z

Convert XOR operation to addition and multiplication:

(22) aux=x+y−2xy

(23) result=x+y−2xy+z−2(x+y−2xy)z

The RICS constraint is:

(24) A=2·aux

(25) B=z

(26) C=result−aux−z

Further, for the XOR operation X⨁Y⨁Z of three 32-bit message words, it is only necessary to use the message word-splitting circuit to split and constrain each message word and generate the circuit for the message bits at the corresponding position. Finally, the calculated message bits are combined into a single message word output through the message word combining circuit.

- Modulo operation circuit

When calculating the intermediate variables SS1, TT1 and TT2, the compression function of SM3 performs a modulo $2^{32}$ addition operation, which leads to the establishment of a constraint relationship between the effective bits before and after the modulo. For the modulo operation in the modulo circuit, the message word splitting operation is used to convert it into message bits, and then the valid bits are constrained to be equal.

For instance, given the message word $X^{\prime }={x_{n+i}}^{\prime }\cdots {x_{n}}^{\prime }\cdots {x_{0}}^{\prime }$ and $X=x_{n}x_{n-1}\cdots x_{0}$:

(27) $$X=X^{\prime }\;\mathrm{mod}\;2^{n}$$

Convert modulo operation to addition and multiplication:

(28) $$X^{\prime }=2^{n}{x_{n+i}}^{\prime }+\cdots +2^{n}{x_{n}}^{\prime }+\cdots +{x_{0}}^{\prime }$$

(29) $$X=2^{n}x_{n}+2^{n-1}x_{n-1}+\cdots +x_{0}$$

(30) $${x_{n}}^{\prime }=x_{n},{x_{n-1}}^{\prime }=x_{n-1},\cdots ,{x_{0}}^{\prime }=x_{0}$$

- Circular shift circuit

The cyclic shift operation used in the SM3 calculation process is to cyclically shift the message word to the right; namely, cyclically shift the original message word $X=x_{n}x_{n-1}\cdots x_{0}$ to the right *i* times to obtain new message word $X^{\prime }$:

(31) $$X^{\prime }=X\gg i$$

Convert shift operation to addition and multiplication:

(32) $${x_{k}}^{\prime }=x_{(k+i)\;\mathrm{mod}\;n}$$

The RICS constraint is:

(33) $$A={x_{k}}^{\prime }$$

(34) B=1

(35) $$C=x_{(k+i)\;\mathrm{mod}\;n}$$

#### 5.1.2. Core Operation Circuit

- Boolean function FF circuit

The Boolean function FF is a piecewise function, which corresponds to the 64-round function of SM3. Its X⨁Y⨁Z part uses the three-digit XOR circuit in the auxiliary operation circuit described above for conversion; and for the operation (X∧Y)∨(X∧Z)∨(Y∧Z), it is converted into the bit form x,y,z by message word splitting and merging module in the auxiliary arithmetic circuit. For the single-bit form, the Boolean-converted form is:

(36) result=x∗y+(1−x)∗z

The RICS constraint is:

(37) A=x

(38) B=y−z

(39) C=result−z

Finally, the individual message bits are combined into a complete message word using the Message word merging module in the auxiliary arithmetic circuit.

- Boolean function GG circuit

Similarly, the Boolean function GG is the piecewise function, which corresponds to the 64-round function of SM3. Its X⨁Y⨁Z uses the three-digit XOR circuit in the auxiliary operation circuit described above for conversion. For the operation (X∧Y)∨(¬X∧Z), it is converted into the bit form x,y,z by message word splitting and merging module in the auxiliary arithmetic circuit. For the single-bit form, the Boolean-converted form is:

(40) aux=x+y+z−2∗result

(41) aux∗(1−aux)=0

The RICS constraint is:

(42) A=x+y+z−2∗result

(43) B=1−(x+y+z−2∗result)

(44) C=0

Likewise, the individual message bits are combined into a complete message word using the Message word merging module in the auxiliary arithmetic circuit.

- Permutation function circuit

The permutation function equations used by SM3 involve two operations of cyclic shift and three-digit XOR. Thus, the corresponding permutation function circuit and R1CS constraint can be generated only by combining the circular shift circuit and the three-digit XOR circuit in the auxiliary operation circuit.

#### 5.1.3. Iterative Compression Circuit

- Message expansion circuit

The message expansion calculation includes two iterative forms to expand the message word to 132. For the message expansion calculation $W_{j}=P_{1}\left(W_{j-16}⨁W_{j-9}⨁\left(W_{j-3}\ll 15\right)\right)⨁\left(W_{j-13}\ll 7\right)⨁W_{j-6}$, which can pass from the inside to the outside through the cyclic shift circuit for the three-digit XOR circuit and the permutation function circuit $P_{1}$ of the lower circuit nested combination. For the message expansion calculation ${W_{j}}^{\prime }=W_{j}⨁W_{j+4}$, a three-number XOR circuit can be used, inputting the first two message words and setting the third number to the constant zero.

- Round function circuit

Since each round function performs multiple calculations, each calculation can realize the conversion of the addition and multiplication circuits by multiplexing the underlying circuit. The multiplexing relationship between each calculation expression of the SM3 round function and the sub-circuit modules is shown in Table 2.

#### 5.1.4. Merkle–Damgård Circuit

This circuit is at the top of the circuit layered architecture of this design, and is also the input and output circuit for the SM3 hash preimage zero-knowledge proof. The input variable of this circuit is the SM3 preimage *x*, the output variable is the hash value *y*, and the constraint relationship to be proved is SM3(x)=y. Next, the constraint relationship will be built using the lower-level circuit module to construct a multi-message block computing circuit.

When SM3 preprocesses the message, it will pad the message to make the message length an integer multiple of 512. This step will generate a large number of intermediate variables and constraints between variables, resulting in redundant circuit size, especially when the length to be padded is long.

Since the 64-bit bit string at the end of the padded message is the binary representation of the length of the original message, the original message can be directly restored through the padded message. Due to the fact that this process is not part of a one-way function, implementing message stuffing outside the circuit without including this process into the zero-knowledge proof circuit does not compromise security.

After the message padding preprocessing is completed, the message is divided into blocks $B_{0},B_{1}\cdots B_{n}$ according to the size of 512 bits. For the SM3 calculation using the Merkle–Damgård structure, the corresponding constraint for each message block is $V_{i+1}=CF\left(V_{i},B_{i}\right)$. Based on this basic calculation unit, the constraint relation SM3(x)=y can be transformed into the constraints of the image and preimage of *n* compression functions CF:

(45) $$\begin{array}{cc} V_{1}= & CF(V_{0},B_{0})\\ V_{2}= & CF(V_{1},B_{1})\\ \; & \cdots \\ y= & CF(V_{n},B_{n}) \end{array}$$

Where CF is the compression function and $V_{0}$ is the initial value of registers A∼H.

Finally, for the constraint of a single compression function, it contains 116 rounds of message expansion and 64 rounds of round functions, which can be realized by splicing the message expansion circuit and the round function circuit in the iterative operation circuit layer.

### 5.2. Circuit Implementation Paradigm

Regarding the realization of specific circuits, there are two types of general-purpose circuit generator implementations and dedicated implementations. The former only need to output a C program with formatted input and output description arguments to a circuit generator to automatically generate a circuit in the form of R1CS, such as the circuit generator Pinocchio. In contract, after designing the circuit structure manually, we realized the sub-circuit modules one by one from the bottom up.

The code implementation of this paper is based on the libsnark framework. The libsnark code library provides various circuit base classes, and each base class module implements the member methods of circuit instantiation, arithmetic constraint binding and internal state calculation. Therefore, all circuit modules in this paper will inherit these base classes for implementation.

Each circuit type inherits from the circuit module base class. Its member variables include sub-circuit objects and intermediate variable objects. Sub-circuit objects refer to all sub-circuits that can be reused by the current circuit type. Intermediate variable objects refer to all intermediate variables not included in the sub-circuit in the current circuit calculation process. Its member methods are circuit constructor, constraint generation function and state calculation function; corresponding to the circuit generation paradigms are circuit instantiation, arithmetic constraint binding, and internal state calculation.

#### 5.2.1. Circuit Instantiation

Circuit instantiation refers to generating circuit instances for each intermediate variable of a computational process. For each zk-SNARKs proof, libsnark will initialize a blank circuit board object. The intermediate variables corresponding to each circuit line in the circuit board belong to the specified finite field, and each intermediate variable has a unique label on the circuit board.

The circuit instantiation step is carried out in the constructor of the circuit object, which takes the input and output variables of the current circuit as function parameters to construct the variables inside the circuit and creates unique variables for these internal variables on the entire proof board label. Starting from the input variable of the circuit, after each round of calculation, the newly generated intermediate value of the calculation needs to be allocated a unique variable in the circuit board. If the variable requires message word splitting operation, the corresponding bit variable needs to be allocated additionally.

#### 5.2.2. Arithmetic Constraint Binding

After the circuit is instantiated, all intermediate variables of the current zero-knowledge proof calculation are bound to the corresponding wires in the circuit with unique labels, and these wires are independent of each other. The arithmetic constraint binding step is to constrain these wires to each other through the multiplication gate and the addition gate. This step only needs to input the allocated circuit variables according to the R1CS constraint form given in Section 3 into the add constraint interface provided by libsnark, which can bind these variables to specified constraints.

Add R1CS constraint form steps as:

(1) According to the previous conversion results, extract the three parts (*A*, *B*, and *C*) of the R1CS constraint form, such as Equations (13) to (15);
(2) *A*, *B*, and *C* contain the addition and multiplication of one or more variables, called linear combinations of circuit variables. In the implementation process, these variables are formed into corresponding linear combination objects through the interface provided by libsnark;
(3) Input the linear combination objects $A_{LC}$, $B_{LC}$, and $C_{LC}$ as the parameters of the R1CS constraint interface added in libsnark to generate corresponding constraint object.

#### 5.2.3. Internal State Calculation

Only if the prover has the correct input message can all intermediate variables in the SM3 execution process be calculated correctly. Thus, all the R1CS constraints generated by the arithmetic constraint binding process are satisfied, and a correct proof is provided.

Any sub-circuit only exposes the circuit input and output but hides the internal variables. The internal state calculation process is to assign the input variable of the circuit and then calculate the value of the internal variable step by step according to the calculation process of the circuit. Then, we assign it to the internal variable set in the previous step until the entire calculation process is completed.

There are two points to note. First, the internal state calculation method only depends on the calculation itself. If the calculation itself is a mixed operation, it can be directly implemented by the corresponding operator without considering the conversion to addition and multiplication. Second, attention should be paid to the form in which the internal variables exist. If the input is an integer on a finite field, and the circuit variables exist in the form of bits, the corresponding conversions should be performed.

### 5.3. Circuit Implementation Optimization

Under the premise that the circuit conversion process is correct, it is necessary to optimize the circuit implementation further, reduce circuit redundancy, and improve the proof efficiency. Hence, three main optimizations have been made in the specific implementation process.

#### 5.3.1. Avoid Complex Control Flow

The SM3 algorithm is an arithmetic-based algorithm that does not involve complex control flow or memory access. If the Pinocchio circuit generator automatically analyzes the C program and generates the circuit, it will generate a large number of redundant variables on some control flow statements. A constraint circuit will be generated between the redundant variables, resulting in the circuit being too large. Consequently, the implementation process of this paper actively avoids the characteristics of high-level programming language control flow and memory access, as a result of which the coding process is mainly based on assignment, loop, and conventional operations.

#### 5.3.2. Reuse Temporary Variables

In different life cycles of program execution, the same intermediate calculation value may be assigned to different temporary variables. Multiple circuit variables will be assigned to the circuit after analysis by the general zero-knowledge proof circuit generation tool. The paper binds these temporary variables that represent the same intermediate value in different life cycles to the libsnark circuit board as a globally unique circuit variable, which realizes the reuse of temporary variables.

#### 5.3.3. Preprocess Message Padding

In this paper, the message padding step is carried out outside the zero-knowledge proof circuit to avoid a large amount of circuit redundancy due to the variable-length variable allocation and constraint operation of message padding. In the implementation process, a program unrelated to the zero-knowledge proof circuit will be used to pad the input message according to the SM3 padding rule to generate a message block with multiple 512 bits, which will be used as the fixed-length input of the SM3 zero-knowledge proof circuit.

## 6. Test and Analysis

### 6.1. Test Environment

This paper tests the implemented SM3 zero-knowledge proof circuit from two perspectives of functional and performance. The test uses the Ubantu operating system and the libsnark zero-knowledge proof framework regarding the physical machine test environment. Moreover, we use Pinocchio as a contrast circuit generator.

The specific physical machine test environment is shown in Table 3.

### 6.2. Functional Test

The functional test is used to test whether the SM3 zero-knowledge proof circuit is implemented correctly, and it is also the most basic test. Likewise, the correctness of the zero-knowledge proof is an essential part of its security; hence, this functional test also verifies the security of the SM3 zero-knowledge proof circuit implementation from the perspective of correctness.

Typically, *y* is the public input of the zero-knowledge proof system, and *x* is the secret input. We randomly select the input message *x*, and calculate the SM3 value y=SM3(x). The prover holds the message $x^{\prime }$, generates about $y=SM3(x^{\prime })$ through the interface implemented in this paper, and completes the zero-knowledge proof $x^{\prime }=x$. The verifier inputs the zero-knowledge proof obtained from the prover and the public input *y* to be verified through the interface. The verification program is run to obtain the verification result.

**Figure 5:**
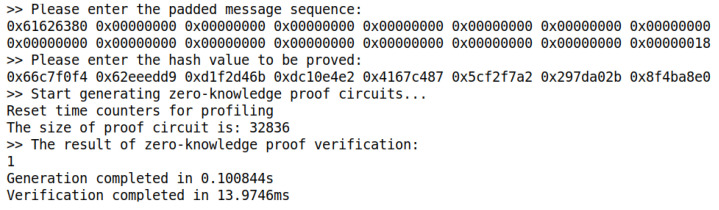
The program output of test case 1.

**Figure 6:**
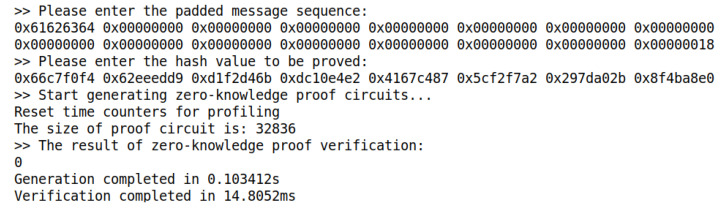
The program output of test case 2.

The correctness of the SM3 zero-knowledge proof circuit is verified by changing the secret input in the test. Table 4 selects six sets of test cases from 1000 tests for display. Figure 5 and Figure 6 show the test cases of test number 1 and test number 2 in Table 4, respectively, and the two are used as a set of test comparisons. Eventually, the 1000 sets of tests prove to be 100% correct.

### 6.3. Performance Test

This article provides performance tests in both longitudinal and horizontal dimensions. The longitudinal test is used to compare the circuit size gap between the efficient implementation of the SM3 zero-knowledge proof circuit and the general zero-knowledge proof circuit generation tool Pinocchio, which reflects the performance difference between the designed circuit structure and the automatically generated circuit structure. The performance of our SM3 scheme is reflected by comparing the efficiency of manual generation of the SHA256 hash function circuit. The horizontal test is used to compare the circuit size gap between this implementation and the most efficient implementation of the SHA256 zero-knowledge proof circuit. The horizontal test compares the circuit design efficiency based on the calculation process of the hash function itself.

**Figure 7:**
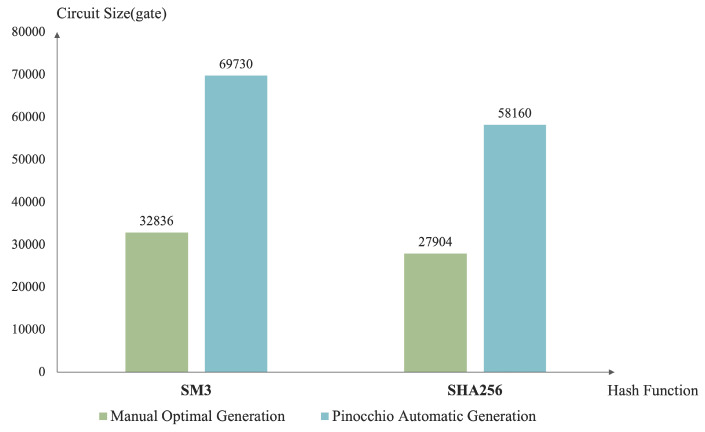
The comparison results of the SM3 circuit performance test.

The performance test results are shown in Figure 7. A total of four sets of data are tested, representing the optimal manual generation and the automatic generation of the circuit size by Pinocchio of SM3 and SHA256.

It can be seen from the longitudinal test results that the size of the circuit implemented in this paper is about half of the size of the SM3 circuit generated by the general-purpose zero-knowledge proof circuit generation tool Pinocchio. Analyzed in principle, Pinocchio will generate too many repeated intermediate variables during the conversion process, and constraining these repeated intermediate variables will cause a lot of redundancy [36]. The manual circuit generation process reduces repetitive variables and improves circuit generation efficiency.

According to current results from industry, the optimal practice of the SHA256 zero-knowledge proof circuit generates 27,904 circuit gates, while the number of circuit gates generated by the automatic circuit generation tool reaches 58,160 [37]. Both the implementation of SM3 in this paper and the rest of the research SHA256 is achieved via manual circuit generation to reduce intermediate variable redundancy and reduce circuit size. Taking SHA256 as the comparison object, it can be seen from the horizontal test results that the optimal practice of SHA256 reduces the circuit size by half, which is comparable to the optimization efficiency of this paper.

SM3 and SHA256 have certain similarities in iterative structure and some logical functions. The reason why SM3 is larger than SHA256 circuit is that, on the one hand, SM3 generates more than 132 message words than SHA256 through message expansion to increase the complexity; on the other hand, the iterative compression step of SM3 involves more mixed operations, requiring frequent message word splitting and merging operations.

Moreover, Poseidon is a hash function specially designed for zero-knowledge proof calculations. As can be seen from Table 5, although the size of SM3 is reduced to nearly 30,000 in this paper, the size of the final circuit generated by Poseidon is only a few hundred gates due to the use of a simple Substitution box (S-box). However, a SNARK-friendly hash function such as Poseidon cannot reach the strength of SM3 and SHA256 in terms of security; as aresult, this SM3 zero-knowledge proof circuit implementation is irreplaceable for scenarios with high security requirements.

Correspondingly, we also test the generation and verification time of the zero-knowledge proof circuit. This paper mainly focuses on designing and implementing the zero-knowledge proof circuit based on SM3 and completes the zero-knowledge proof process through a fixed back-end library. The current industrial zero-knowledge proof generation time is in the order of seconds, while the verification time is in the order of milliseconds. According to Figure 5 and Figure 6 above, when the hash input is short, the zero-knowledge proof verification time of SM3 circuit designed in this paper is about 14 ms, which is in line with the industry average. Since this paper focuses on testing the SM3 circuit, the implementation is prefilled, and there is no CRS trusted setup and other links. Therefore, the generation time of SM3 circuit in the zero-knowledge proof generation is 103 ms in this paper with high efficiency.

**Figure 8:**
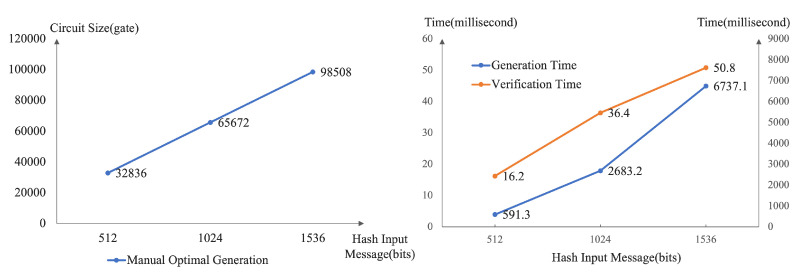
The Performance of circuit size, generation time and verification time under different sizes of hash input messages in tests.

Further, after multiple tests, we show that the circuit size and zero-knowledge proof time increase linearly with the input message’s length in Figure 8. The SM3 algorithm requires that the input message be padded to a multiple of 512 bits. Only one message block is needed when the hash input is a short message and the input message is less than 512 bits. At this time, the SM3 circuit size is 32,836. As the input message’s length grows, the message needs to be padded into multiple message blocks. In the implementation, multiple SM3 circuits are connected in series, multiplying the total circuit size, generation and verification time.

To summarize, this SM3 zero-knowledge proof circuit reduces the circuit size by half compared to the automatic circuit generation tool. In comparison, half of the performance improvement is equivalent to the best practice efficiency of the SHA256 zero-knowledge proof circuit, which proves the excellent performance of the scheme from the side. When the proof time complexity of the zero-knowledge proof back-end protocol is O(nlogn), the circuit optimization effect will further reduce the zero-knowledge proof time by more than half.

## 7. Application Design and Implementation of Zero-Knowledge Proof Based on SM3

Cryptography is one of the cornerstones of blockchain, and cryptography concepts are widely used in blockchain. This section introduces several zero-knowledge proof protocols that combine elliptic curve discrete logarithms and hash chains. These efficient zero-knowledge proof protocols based on SM3 can improve the overall operating efficiency and security of the system in the blockchain. Both these protocols use the libsnark library for programming based on the realized SM3 zero-knowledge proof circuit, and follow the above circuit generation paradigm for programming.

### 7.1. Proof of Equivalence between SM3 and Elliptic Curve Discrete Logarithm

Since the elliptic curve discrete logarithm cryptosystem can provide more robust security under the same key length, many cryptographic algorithms based on the elliptic curve discrete logarithm problem have a wide range of practical applications. For instance, the electronic signature algorithms are implemented by elliptic curve ECDSA and EdDSA, the ElGamal Encryption Algorithm, and the Hybrid Encryption Scheme based on Elliptic Curve and Pedersen commitments in the form of elliptic curves, etc. The elliptic curve signature algorithm accounts for an important proportion in the blockchain, and ECDSA appears in many famous blockchain projects, such as Bitcoin and Ethereum.

These algorithms all use elliptic curves (secp256k1, curve25519, or p521) for calculation, their private key is an integer, and the public key is the corresponding point on the elliptic curve. Combining elliptic curve discrete logarithms with SM3 can construct more practical privacy-preserving protocols.

#### 7.1.1. Protocol Design

As mentioned above, the protocol is programmed with the libsnark library, and its steps are as follows.

(1) The prover selects an integer from 0∼p−1 as the secret number *x* to be proved;
(2) The prover multiplies the secret number *x* by the base point of the elliptic curve $E_{p}\left(a,b\right)$ to calculate the point $y_{E}$ on the elliptic curve;
(3) The prover calculates the SM3 hash value $y_{SM3}=SM3\left(x\right)$ of the secret number *x*;
(4) The prover adopts a general zero-knowledge proof scheme, taking *x* as the secret input, $y_{E}$ and $y_{SM3}$ as the public input.

Then, the prover generates the proof $Proof=ZK\{x|y_{E}=xB\cap y_{SM3}=SM3\left(x\right)\}$.

The verifier runs the polynomial time verification algorithm of the zero-knowledge proof scheme to verify the proof’s correctness.

#### 7.1.2. Protocol Implementation

The protocol implementation steps are as follows.

(1) Implement the message padding algorithm of SM3 outside the circuit, i.e., convert the private input into bit form, and allocate additional bit variables to 512-bit integer multiples on this basis;
(2) Use the padded bit sequence as the input of the SM3 circuit;
(3) Use the unfilled original bit sequence as the input of the elliptic curve calculation;
(4) In the elliptic curve calculation circuit, a message word merging circuit is used to combine the input bit sequence into an elliptic curve calculation unit, and an appropriate elliptic curve is selected to calculate the points on the elliptic curve;
(5) Take the SM3 value to be proved and the elliptic curve point as the output value of the two circuits, respectively;
(6) Constraints and intermediate variable values are generated for the complete circuit according to the circuit generation paradigm, and a zero-knowledge proof is obtained.

This paper finally selects the 256-bit elliptic curve sm2p256v1 in the prime number field in the SM2 national secret elliptic curve encryption [38] as the standard, and its parameters are: p=FFFFFFFEFFFFFFFFFFFFFFFFFFFFFFFFFFFFFFFF00000000FFFFFFFFFFFFFFFF, a=FFFFFFFEFFFFFFFFFFFFFFFFFFFFFFFFFFFFFFFF00000000FFFFFFFFFFFFFFFC, b=28E9FA9E9D9F5E344D5A9E4BCF6509A7F39789F515AB8F92DDBCBD414D940E93.

#### 7.1.3. Test and Analysis

We carry out the functional test of implementing the SM3 zero-knowledge proof circuit. The results show that the verification fails when any of the elliptic curve number multiplication relationships and the SM3 calculation relationship between the secret number input in the test case and the public input are not satisfied. Thus, the protocol is correctly implemented.

Likewise, we carry out the performance test of implementing the SM3 zero-knowledge proof circuit. When the secret number padding length is 512 bits, the circuit size is 33,796. It can be seen from the protocol design that the circuit complexity mainly depends on the SM3 circuit. The part of the elliptic curve discrete logarithm proof is relatively small, and the generation and verification time are also close to the SM3 circuit. After analysis, the generation time is about 700 ms, and the verification time is about 17 ms. Therefore, this paper’s optimization of the SM3 circuit will significantly improve the efficiency of this type of protocol implementation.

### 7.2. Hash Chain Proof Based on SM3

Hash chains have a wide range of applications in key generation and blockchain. For instance, the hash chain uses the one-way characteristic of the hash function to generate a one-time key. The user and the server are verified by the adjacent output of the hash chain. After the verification, the key is updated to the last output on the hash chain. In addition, according to the seriality of the hash chain calculation process, it can be used as a weakly verifiable delay function [39], which can be applied to time-constrained blockchain scenarios such as space–time proofs.

#### 7.2.1. Protocol Design

For a hash chain with a hash function of SM3 and a length of *n*, the prover needs to prove that it holds the input message *x* so that a specific public value $y=SM3^{n}\left(x\right)$ is established, and other information about *x* is not leaked.

Similar to the hash preimage proof, this hash chain proof only contains one private input and one public input, which are the message *x* and the public value *y*, respectively. The prover executes the general zero-knowledge proof algorithm to prove $Proof=ZK\{x|y=SM3^{n}\left(x\right)\}$. The verifier runs the polynomial time verification algorithm of the zero-knowledge proof scheme to verify the proof’s correctness.

#### 7.2.2. Protocol Implementation

Since the topology of the hash chain is chain-like, its programming implementation is relatively simple, and the protocol can be implemented only by connecting the SM3 circuits above in series.

#### 7.2.3. Test and Analysis

We perform multiple sets of tests on this circuit implementation. When the input message’s length is 512 bits, the length of the hash chain is adjusted for comparison. When the hash chain length is one, the circuit degenerates into an SM3 preimage proof circuit with a size of 32,836; when the hash chain length increases to *n*, the circuit size is 32,836*n*, which is consistent with the theoretical analysis size. The performance of the circuit mainly depends on the performance of the SM3 circuit, and both the proof generation and verification time are proportional to the SM3 according to the hash chain length.

## 8. Conclusions

This paper summarizes the current usage scenarios and implementation challenges of SM3 hash preimage zero-knowledge proof, studying the general zero-knowledge proof protocol and the generation technology of a zero-knowledge proof circuit. Moreover, we analyze the basic process and primary consideration factor for implementation of SM3 zero-knowledge proof circuit. On this basis, a four-layer SM3 layered circuit structure is designed, and an elaborate scheme is given for the conversion process of the sub-circuits in each layer. After that, based on the libsnark framework, we implement all sub-circuit modules recursively from top to bottom in accordance with the designed circuit implementation paradigm. Accordingly, we entirely realize the SM3 zero-knowledge proof circuit, which successfully passes the functional and performance tests. In addition, we extract several standard privacy protection requirements in the blockchain field and design an extended zero-knowledge proof protocol combined with SM3.

Although the current research on zero-knowledge proof mainly focuses on the back-end protocol’s efficiency and security, it is always a tedious task to convert the problem to be proved into a zero-knowledge proof circuit. Admittedly, it is impossible for users of zero-knowledge proof technology to manually implement corresponding zero-knowledge proof circuits for all NP problems. Therefore, it is hoped that in the future development of this field, researchers will further investigate automatic conversion tools with high conversion efficiency to reduce circuit redundancy and bring the circuit size close to the theoretical limit.

## Figures and Tables

**Table 1:** This table shows the relevant data of the proof efficiency of several zero-knowledge proof protocols, where *n* refers to the circuit size and *l* refers to the number of variables.

Proof Protocol	Proof Time	Verification Time	Proof Size
Groth16 [2]	O(nlogn)	O(l)	O(1)
Bulletproofs [4]	O(nlogn)	O(nlogn)	O(logn)
zk-STARKs [5]	O(npolylogn)	O(polylogn)	$O({\left(\log n\right)}^{2})$
Sonic [6]	O(nlogn)	O(l+logn)	O(1)
Plonk [35]	O(nlogn)	O(l)	O(1)

**Table 2:** This table shows the multiplexing relationship between the SM3 round function calculation expression and the sub-circuit modules.

Calculation Formula	Sub-Circuit Relationship
$SS1\leftarrow (\left(A\ll 12\right)+E+\left(T_{j}\ll J\right))\ll 7$	Circular shift circuit, Modulo operation circuit
SS2←SS1⨁(A≪12)	Permutation function circuit, Circular shift circuit
$TT1\leftarrow FF_{j}\left(A,B,C\right)+D+SS2+{W_{j}}^{\prime }$	Modulo operation circuit, Boolean function FF circuit
$TT2\leftarrow GG_{j}\left(E,F,G\right)+H+SS1+W_{j}$	Modulo operation circuit, Boolean function GG circuit
C←B≪9,G←F≪19	Circular shift circuit
$E\leftarrow P_{0}\left(TT2\right)$	Permutation function circuit

**Table 3:** The specific physical machine test environment.

Operating System	Ubuntu 20.04 focal
Translater	gcc 9.3.0
Zero-knowledge Proof Framework	libsnark @2af4402
Circuit Generator	Pinocchio v0.5.3

**Table 4:** The inputs and results of 6 sets from 1000 test cases.

Secret Input	Input Size	Public Input	Verification Result
abc	3 bytes	66c7f0f4 62eeedd9 d1f2d46b dc10e4e2 4167c487 5cf2f7a2 297da02b 8f4ba8e0	True
abcd	4 bytes	66c7f0f4 62eeedd9 d1f2d46b dc10e4e2 4167c487 5cf2f7a2 297da02b 8f4ba8e0	Fause
cyperspace	10 bytes	428a7309 6fe62d08 59a42c09 75e659eb c5adce1d de9af1dc 3e0fa80d fd054170	True
securityspace	13 bytes	428a7309 6fe62d08 59a42c09 75e659eb c5adce1d de9af1dc 3e0fa80d fd054170	Fause
teeth	5 bytes	afb4d865 380cfb6a 207a65e7 12cb9bb9 93058d51 02a44269 c2d6a41e dfe973fb	True
tooth	5 bytes	afb4d865 380cfb6a 207a65e7 12cb9bb9 93058d51 02a44269 c2d6a41e dfe973fb	Fause

**Table 5:** The gate count of the typical hash function Poseidon with different inputs and outputs.

Output Length	S-Box	Input Length (Bits)	Elliptic Curve	Circuit Size (Gates)
Poseidon-80	$x^{5}$	510	BLS/BN/Ed	171
Poseidon-128	$x^{5}$	1020	BLS/BN/Ed	300
Poseidon-256	$x^{5}$	1020	BLS/BN/Ed	504

## Data Availability

Not applicable.
